# Organizational Conspiracy Beliefs: Implications for Leadership Styles and Employee Outcomes

**DOI:** 10.1007/s10869-015-9428-3

**Published:** 2015-12-21

**Authors:** Jan-Willem van Prooijen, Reinout E. de Vries

**Affiliations:** 1 Department of Social and Organizational Psychology, VU Amsterdam, van der Boechorststraat 1, 1081BT Amsterdam, The Netherlands; 2 The Netherlands Institute for the Study of Crime and Law Enforcement (NSCR), Amsterdam, The Netherlands; 3 University of Twente, Enschede, The Netherlands

**Keywords:** Organizational conspiracy beliefs, Leadership, Job insecurity, Employee outcomes

## Abstract

**Purpose:**

Belief in conspiracy theories about societal events is widespread among citizens. The extent to which conspiracy beliefs about managers and supervisors matter in the micro-level setting of organizations has not yet been examined, however. We investigated if leadership styles predict conspiracy beliefs among employees in the context of organizations. Furthermore, we examined if such organizational conspiracy beliefs have implications for organizational commitment and turnover intentions.

**Design/Methodology/Approach:**

We conducted a survey among a random sample of the US working population (*N* = 193).

**Findings:**

Despotic, laissez-faire, and participative leadership styles predicted organizational conspiracy beliefs, and the relations of despotic and laissez-faire leadership with conspiracy beliefs were mediated by feelings of job insecurity. Furthermore, organizational conspiracy beliefs predicted, via decreased organizational commitment, increased turnover intentions.

**Implications:**

Organizational conspiracy beliefs matter for how employees perceive their leaders, how they feel about their organization, and whether or not they plan to quit their jobs. A practical implication, therefore, is that it would be a mistake for managers to dismiss organizational conspiracy beliefs as innocent rumors that are harmless to the organization.

**Originality/Value:**

Three novel conclusions emerge from this study. First, organizational conspiracy beliefs occur frequently among employees. Second, participative leadership predicts decreased organizational conspiracy beliefs; despotic and laissez-faire leadership predict increased organizational conspiracy beliefs due to the contribution of these destructive leadership styles to an insecure work environment. Third, organizational conspiracy beliefs harm organizations by influencing employee commitment and, indirectly, turnover intentions.

Belief in conspiracy theories is a widespread societal phenomenon. Large portions of ordinary citizens believe that influential and harmful events—such as economic crises, natural disasters, and wars—are caused by evil conspiracies of powerful individuals or groups (Oliver and Wood 2014; Sunstein and Vermeule 2009). Throughout the social sciences this phenomenon has been subject to extensive research in recent years (for overviews, see Bilewicz et al. 2015; Van Prooijen and Van Lange 2014). These research efforts produced a wealth of findings, empirically linking the tendency to believe in conspiracy theories to detrimental health choices (Thorburn and Bogart 2005), decreased civic virtue (Jolley and Douglas 2014), disagreeableness (Swami et al. 2011), and radicalization (Van Prooijen et al. 2015; see also Inglehart 1987). This stream of research hitherto focused mainly on macro-level conspiracy theories, which are conceptualized as suspicious beliefs about geopolitical decision-making—typically implicating powerful politicians, stigmatized ethnic groups, or entire branches of industry (e.g., “the oil industry,” or “the pharmaceutical industry”). In the present paper, however, we propose that conspiracy beliefs are also likely to emerge in the dynamic, micro-level setting of organizations: Frequently, employees may be suspicious of the possibility that their managers conspire in secret to reach evil goals.

We define such *organizational conspiracy beliefs* as explanatory beliefs among employees who suspect their managers, supervisors, or colleagues to meet in secret in order to achieve goals that are widely seen as malevolent (for related definitions, see Bale 2007; Zonis and Joseph 1994). For instance, in the face of challenging times—such as mergers, acquisitions, economic crises, or organizational downsizing—employees may suspect that their management team has agreed upon a hidden agenda to harm employees’ interests in order to gain additional wealth for themselves. Conspiracy beliefs are conceptually different from distrust: Whereas distrust refers to an abstract aversive feeling toward a person or group, a conspiracy theory is a specific, concrete, and seemingly coherent allegation of misconduct committed by a powerful group of authorities. Correspondingly, a seminal study by Goertzel (1994) found a significant but moderate correlation between trust and societal conspiracy beliefs (*r* = −.37), suggesting that trust and conspiracy beliefs are related but distinct constructs. Likewise, it has been argued and found that conspiracy theories are rooted in the dynamic interplay of multiple factors: Conspiracy theories not only reflect distrust, but also serve as a means to simplify and understand a complex and distressing reality, to make attributions for one’s own disadvantaged position, and to ventilate one’s anger (Abalakina-Paap et al. 1999).

Empirical research thus far had only limited attention for the predictors of such organizational conspiracy beliefs. The concept of organizational conspiracy beliefs is a specific form of the more general conceptualization of suspicion by Bobko and colleagues (Bobko et al. 2014a, b), and the closely associated concept of paranoia (Fenigstein and Vanable 1992; Kramer 1998): Whereas people can be suspicious of the motives or actions of a single individual, conspiracy beliefs by definition are suspicions about secret activities of a group of powerful actors. Nevertheless, suspicion and conspiracy beliefs are rooted in comparable psychological processes: Both constructs are associated with increased cognitive activity (that is, sense-making efforts), uncertainty, and attributions of malevolent intent to the implicated actors. There are thus strong conceptual links between the constructs of suspicion and conspiracy beliefs.

In the present study, we seek to explore the role of conspiracy beliefs in an organizational setting. We specifically endorse a three-step approach. First, we examine to what extent organizational conspiracy beliefs can be predicted by both destructive leadership styles (i.e., despotic and laissez-faire leadership) and constructive leadership styles (i.e., participative and charismatic leadership). People frequently regard their leader as representative for the entire organization (Tyler and Blader 2003; see also Van Prooijen et al. 2004), and hence, the behavior of leaders may be regarded as diagnostic for the likelihood of foul play within the organization, in the form of malevolent conspiracies. Second, we examine a hypothesized mediator of the relationship between these leadership styles and organizational conspiracy beliefs. Integrating the literature on belief in conspiracy theories and leadership, we predict a central role for feelings of job insecurity (e.g., Ashford et al. 1989; De Witte 2005). Third, and finally, we explore whether organizational conspiracy beliefs have implications for employee outcomes. We specifically focus on the extent to which employees feel committed to the organization (e.g., Meyer and Allen 1991), and the extent to which they plan to resign their job (i.e., turnover intentions; Stiglbauer et al. 2012). In the following, we first introduce general insights into the psychology of conspiracy beliefs. Then, we extrapolate these insights to an organizational context, and distil our hypotheses.

## Leadership and Organizational Conspiracy Beliefs

One pertinent finding is that a primary predictor of belief in one conspiracy theory is belief in a different, unrelated conspiracy theory (e.g., Abalakina-Paap et al. 1999; Goertzel 1994; Lewandowski et al. 2013; Swami et al. 2010, 2011, 2013; Wood et al. 2012). This suggests that, whereas the content of various conspiracy theories may differ enormously, belief in such theories is grounded in an underlying conspiratorial mindset that can be predicted by a range of dispositional and contextual factors. A core theoretical insight is that such a conspiratorial mindset is activated particularly in uncertain, fearful, or threatening situations. Early writings by Hofstadter (1966) already explicated that belief in conspiracy theories is fueled by a desire to explain distressing events that are hard to explain otherwise, especially among citizens who feel powerless or voiceless. More generally, belief in conspiracy theories has been associated with the human desire to make sense of the social world. Uncertain, threatening events have been found to prompt mental sense-making processes, designed to promote understanding of the event (e.g., Park 2010). Such sense-making is at the core of paranoia (Kramer 1998), suspicion (Bobko et al. 2014a, b), and belief in conspiracy theories (Bale 2007; Shermer 2011).

Empirical research supports this influence of distressing, uncertainty-eliciting events on belief in conspiracy theories. For instance, research reveals that influential, harmful events (e.g., a president is assassinated) lead to stronger conspiracy beliefs than events that are less influential or harmful (e.g., the assassination attempt fails; McCauley and Jacques 1979). Such consequence-cause matching in conspiracy beliefs has been found to be attributable to people’s sense-making motivation (Van Prooijen and Van Dijk 2014). Moreover, people believe more strongly in conspiracy theories when they generally experience a lack of control (Van Prooijen and Acker 2015; Whitson and Galinsky 2008; see also Sullivan et al. 2010). Finally, the experience of subjective uncertainty predicts the psychological processes underlying belief in conspiracy theories (Newheiser et al. 2011; Van Prooijen, in press; Van Prooijen and Jostmann 2013). All in all, there is strong consensus in the research literature that conspiracy beliefs gain momentum particularly in adverse, uncertain social circumstances.

These considerations are relevant to predict empirical relationships between leadership and organizational conspiracy beliefs, as leaders have an impact on how adverse employees experience their work environment, and the corresponding extent to which they feel uncertain about their jobs. Leadership is one of the most frequently studied topics within the organization sciences (Bass and Bass 2009). A primary focus within this research domain is the question what leader traits and behaviors determine leader effectiveness (e.g., DeRue et al. 2011; De Vries et al. 2002). Such features of leaders are usually conceptualized into various styles of leadership, that can be distinguished using a number of dimensions, two of which have received a lot of empirical support (De Vries 2008; Redeker et al. 2014), specifically how active versus passive a leader operates (also referred to as the agency, control, or dominance dimension) and how supportive (or: constructive) versus unsupportive (or: destructive) a leader acts toward subordinates (also referred to as the communion, love, or affiliation dimension). In the present contribution, we focus on the four leadership styles that represent these dimensions, namely despotic leadership (destructive/active), laissez-faire leadership (destructive/passive), charismatic leadership (constructive/active), and participative leadership (constructive/passive)^1^ (Redeker et al. 2014).

### Destructive Leadership Styles

How are the two destructive styles associated with organizational conspiracy beliefs? Despotic leaders—that is, leaders who behave in an authoritarian, harsh manner toward employees, and do not easily accept criticism—are insensitive to the needs of employees, and have been associated with perceptions of abusive supervision (Kiazad et al. 2010; Martinko et al. 2013). As such, despotic leaders contribute to a work environment where employees feel dominated, controlled, and marginalized. Such feelings of marginalization have consequences for how employees experience their job: Specifically, despotic leaders are likely to elicit feelings of job insecurity among employees, as such leaders provide little confidence that they will try to retain an employee’s position in the face of organizational change (e.g., Padilla et al. 2007). In a somewhat different fashion, laissez-faire leaders also contribute to an insecure work environment. They are characterized by a lack of leadership, and do not intervene unless it is absolutely necessary. Laissez-faire leaders are hence considered indifferent, and consequently, it may be hard for employees to establish how well they perform on their tasks, or how much their leader respects them. Laissez-faire leadership has indeed been found to be detrimental to leader effectiveness (DeRue et al. 2011), and correspondingly, it stands to reason that laissez-faire leaders increase employees’ feelings of job insecurity.

In sum, although through different types of behaviors, despotic and laissez-faire leadership styles both contribute to a workplace where employees feel insecure about their jobs. Integrating this insight with the notion that feelings of uncertainty stimulate belief in conspiracy theories (Sullivan et al. 2010; Van Prooijen, in press; Van Prooijen and Acker 2015; Van Prooijen and Jostmann 2013; Whitson and Galinsky 2008), it can be expected that these aversive leadership styles predict organizational conspiracy beliefs. Specifically, due to their influence on the experience of job insecurity, despotic and laissez-faire leadership stimulate employees to make sense of their leader’s behavior, and of the circumstances that they find themselves in. Building on the literature on conspiracy beliefs reviewed above, it can be predicted that such mental sense-making efforts manifest themselves in conspiratorial perceptions among employees, stipulating that their supervisor could be involved in larger, secret schemes within the organization that are designed to deceive or harm them. Based on this line of reasoning, we formulate the following hypotheses:

#### **Hypothesis 1a**

A despotic leadership style positively predicts organizational conspiracy beliefs.

#### **Hypothesis 1b**

A laissez-faire leadership style positively predicts organizational conspiracy beliefs.

#### **Hypothesis 2**

The relations of despotic and laissez-faire leadership styles with organizational conspiracy beliefs are mediated by increased feelings of job insecurity.

### Constructive Leadership Styles

Whereas destructive leadership styles are expected to increase organizational conspiracy beliefs, constructive leadership styles may decrease such beliefs. It has generally been noted that constructive leadership styles contribute to a positive work environment by decreasing stress and increasing commitment among employees (e.g., Britt et al. 2004; Dale and Fox 2008). These positive effects are evident in both of the constructive leadership styles that are under investigation here, that is, charismatic and participative leadership. Charismatic—or transformational—leaders inspire employees to think and act in the collective interest, and to perceive the organization’s goals as their own goals. For instance, charismatic leaders promote employees’ feeling that their work is important (Bono and Judge 2003), and they make employees feel more empowered in their jobs (Avolio et al. 2004). Of particular relevance for the present purposes, charismatic leaders make employees feel more comfortable when faced with the insecurities associated with organizational change (Herold et al. 2008). This suggests that charismatic leaders may ameliorate feelings of job insecurity. Following the assumed link between job insecurity and organizational conspiracy beliefs outlined above, charismatic leaders should therefore decrease organizational conspiracy beliefs.

Participative leaders, then, solicit the input of their employees by asking for their opinions when important decisions need to be made, and by including them in vital decision-making processes. These consultation behaviors displayed by participative leaders are closely associated with the basic procedural justice strategy of ‘voice’ (Furst and Cable 2008). In a wide variety of social settings, applying procedural justice principles helps people to manage basic uncertainties (Van den Bos and Lind 2002) and improve the relation between leaders and followers (Tyler and Blader 2003; Van Prooijen et al. 2004). These procedural justice effects are also commonly found in the context of organizations (e.g., Brockner et al. 1990; Van Knippenberg et al. 2007). Correspondingly, research reveals positive effects of participative leadership on feelings of empowerment and trust (Huang et al. 2009). These findings suggest empirical relationships between participative leadership, decreased job insecurity, and therefore also decreased organizational conspiracy beliefs. In sum, we had the following predictions for supportive leadership styles:

#### **Hypothesis 3a**

A charismatic leadership style negatively predicts organizational conspiracy beliefs.

#### **Hypothesis 3b**

A participative leadership style negatively predicts organizational conspiracy beliefs.

#### **Hypothesis 4**

The relations of charismatic and participative leadership styles with organizational conspiracy beliefs are mediated by decreased feelings of job insecurity.

## Organizational Conspiracy Beliefs and Employee Outcomes

An additional goal of the present contribution is to investigate the relationship between organizational conspiracy beliefs and employee outcomes, specifically organizational commitment and turnover intentions. Such employee outcomes are associated with leadership styles, and are hence frequently regarded as indicators of leadership effectiveness (e.g., DeRue et al. 2011). Previous research has documented that job insecurity exerts an influence on both these employee outcomes. Notably, job insecurity has been found to decrease organizational commitment, that is, the extent to which employees connect their identity to the organization (Chirumbolo and Hellgren 2003). Likewise, job insecurity increases turnover intention, that is, the intention to quit one’s job in the foreseeable future (Stiglbauer et al. 2012). Little is known, however, about the question whether organizational conspiracy beliefs explain a substantial portion of the variance in these effects of job insecurity on employee outcomes.

In the present study, we investigate whether or not organizational conspiracy beliefs can predict such employee outcomes, and explore the possibility that it mediates the effects of job insecurity. Belief in conspiracy theories has been found to alienate people from the social system that they function in (Abalakina-Paap et al. 1999), and to psychologically and behaviorally disengage them from their leaders (Jolley and Douglas 2014). Extrapolating these insights to an organizational context, employees may be unwilling to connect their identity to the organization if they believe that powerful members representing that organization conspire against them. By the same token, it is likely that employees are more open to the possibility of leaving their organization, to the extent that they believe that their organization is permeated with conspiracies. These considerations suggest that organizational conspiracy beliefs have sizeable implications for employees’ commitment toward the organization, as well as for their intention to quit their jobs. We test the following hypotheses:

### **Hypothesis 5a**

Organizational conspiracy beliefs negatively predict organizational commitment.

### **Hypothesis 5b**

Organizational conspiracy beliefs positively predict turnover intention.

## Method

The study was conducted through the Amazon Mechanical Turk website, where it was advertised as a “Survey regarding leaders at work.” Amazon Mechanical Turk is an internet forum that, in comparison to many other types of samples, often yields more demographically diverse respondents, and at least equally reliable data (Buhrmester et al. 2011). Moreover, many well-established cognitive-behavioral effects replicate on Amazon Mechanical Turk as well as on other samples (Crump et al. 2013). The study lasted about 15–20 min, and participants received a small payment for participation (0.75 US $).

### Participants

All participants were from the US. In the study ad (and in the informed consent), we asked participants to conduct the study only (1) if they have been actively employed for at least 3 months in their current organization; (2) if they have a supervisor; and (3) if their current organization has at least 10 employees. Initial data screening revealed that 7 participants did not meet these criteria; they were excluded from further analyses. The remaining sample contained 193 participants (111 men, 82 women; age range 21–61 years; *M*_age_ = 31.26, SD = 7.78). Participants had a mean tenure in their organization of 5.12 years (SD = 4.31; range 6 months to 30 years), and the median size of the organization that participants worked for was 67 employees (range 10 to an estimated 100,000 employees).

### Measures

Participants responded to all items below on a scale ranging from 1 (*strongly disagree*) to 5 (*strongly agree*).

#### Leadership Measures

To measure *despotic leadership*, we used the 6-item despotic leadership questionnaire (De Hoogh and Den Hartog 2008). Example items are “My supervisor is punitive; has no pity or compassion”; and “My supervisor expects unquestioning obedience of those who report to him/her” (*α* = .91).

To measure *laissez*-*faire leadership*, we used the 7-item passive leadership scale from Den Hartog et al. (1994), which is derived from the Multifactor Leadership Questionnaire (Bass and Avolio 1990). Example items are “My supervisor avoids getting involved in important decisions,” and “My supervisor only takes action when things go wrong” (*α* = .87).

For *charismatic leadership* we utilized 8 items drawn from the charismatic leadership in organizations scale (De Hoogh et al. 2004). Example items are “My supervisor has a vision of the future” and “My supervisor can convince others well of his/her position” (*α* = .79).

Finally, to measure *participative leadership* we used the 6-item power sharing scale (De Hoogh and Den Hartog 2008). Example items are “My supervisor allows subordinates to have influence on critical decisions,” and “My supervisor will reconsider decisions on the basis of recommendations by those who report to him/her.”

#### Job Insecurity

We measured *job insecurity* with two items: “I feel insecure about my position (it is not clear to me if my job will continue to exist),” and “If this organization reorganizes, my job will likely disappear.” These two items were strongly correlated (*r* = .72, *p* < .001), and we averaged them into a composite index of job insecurity.

#### Organizational Conspiracy Beliefs

To measure organizational conspiracy beliefs, we asked participants’ agreement to the following 9 items^2^: “Our management has a hidden agenda,” “Our management had hidden goals which will benefit only them,” “I suspect that our managers frequently lie to employees about important issues.” “Our managers would never consciously hide important information from us employees” (recoded), “Our supervisors would never conspire against subordinates” (recoded), “Our managers gossip about subordinates behind their backs,” “Our supervisors work together to achieve a hidden agenda that they deliberately keep secret,” “Our supervisors pass on confidential data regarding us employees to one another,” and “Our supervisors try to achieve hidden, malevolent goals.” These items were averaged into a reliable scale of organizational conspiracy beliefs (*α* = .87).

#### Employee Outcomes

We measured *organizational commitment* with four items: “I am committed to my organization,” “I identify with my organization,” “My organization is an important part of who I am,” and “I feel I belong in this organization.” These four items were averaged into a reliable scale of organizational commitment (*α* = .86).

Finally, to measure *turnover intentions* we assessed the following two items: “I intend to quit my job in the near future” and “I intend to resign in the next year.” These items were strongly correlated (*r* = .80, *p* < .001) and were hence averaged into an index of turnover intentions. At the end of the questionnaire, participants were thanked and debriefed online.

## Results

The means, standard deviations, and inter-correlations of the study variables are displayed in Table 1. This table reveals that all leadership styles were significantly correlated with organizational conspiracy beliefs. Moreover, conspiracy beliefs were significantly correlated with job insecurity and with both the employee outcomes that are under investigation here (i.e., organizational commitment and turnover intentions). In the following, we first conduct regression analyses to establish to what extent the four leadership styles uniquely predict organizational conspiracy beliefs (Hypotheses 1a, 1b, 3a, and 3b) and job insecurity. After that, we utilize structural equation modeling to examine the mediating role of job insecurity between leadership styles and organizational conspiracy beliefs (Hypotheses 2 and 4), and to establish the extent to which organizational conspiracy beliefs subsequently predict employee outcomes (Hypotheses 5a and 5b).

**Table 1:** Means, standard deviations, and inter-correlations of the study variables

	*M*	*SD*	1	2	3	4	5	6	7	8	9	10	11	12
1. Gender	–	–	–											
2. Age	31.26	7.78	.14	–										
3. Tenure (in years)	5.12	4.31	.07	.62***	–									
4. Organization size	3400	15313	−.00	−.05	−.09	–								
5. Charismatic leadership	3.94	0.53	.13	−.01	.08	−.08	*.79*							
6. Participative leadership	3.63	0.59	.03	.04	.16*	−.04	.62***	*.70*						
7. Despotic leadership	2.85	1.00	−.08	−.18*	−.11	−.01	−.25**	−.45***	*.91*					
8. Laissez-faire leadership	3.02	0.87	−.04	−.15*	−.10	−.04	−.12	−.10	.60***	*.87*				
9. Job insecurity	2.88	1.10	−.06	−.10	−.10	−.02	−.23**	−.24**	.60***	.70***	*.83*			
10. Org. conspiracy beliefs	3.03	0.79	−.03	−.12	−.11	−.01	−.28***	−.50***	.66***	.50***	.55***	*.87*		
11. Turnover intentions	2.82	1.25	−.08	−.19*	−.21**	.06	−.37***	−.37***	.49***	.48***	.55***	.50***	*.89*	
12. Organizational commitment	3.89	0.77	−.10	−.10	.12	−.08	.54***	.56***	−.13	−.00	−.08	−.28**	−.40***	*.86*

Reliabilities are in italics on the diagonal

*N* = 193, * *p* < .05; ** *p* < .01; *** *p* < .001

### Leadership Styles and Conspiracy Beliefs

We analyzed the relation between leadership styles and organizational conspiracy beliefs with a hierarchical regression analysis.^3^ We entered age, gender, tenure in the organization, and size of the organization as control variables in Step 1 of the regression model. In Step 2, we added the four leadership styles as predictors. Organizational conspiracy beliefs was the criterion variable. This analysis indicated that Step 1 was not significant, *F*(4, 187) = 0.86, *p* = .49. Step 2, however, added significantly to the regression model (Δ*R* ^*2*^ = .51), *F*(4, 183) = 49.25, *p* < .001. The full regression model was significant, (*R* ^*2*^ = .53), *F*(8, 183) = 25.51, *p* < .001.

The results are displayed in Table 2. Despotic and laissez-faire leadership styles both were significant positive predictors of organizational conspiracy beliefs, participative leadership was a significant negative predictor of organizational conspiracy beliefs. The effect of charismatic leadership was not significant. These findings support Hypotheses 1a, 1b, and 3b, but they do not support Hypothesis 3a. Employees’ organizational conspiracy beliefs are positively related with despotic and laissez-faire leadership, negatively related with participative leadership, and unrelated with charismatic leadership.

**Table 2:** Organizational conspiracy beliefs and job insecurity as a function of leadership styles

	Organizational conspiracy beliefs				Job insecurity			
	*B*	SE	*β*	*t*(187)	*B*	SE	*β*	*t*(187)
Step 1								
Gender	−.02	.12	−.01	−0.16	−.08	.16	−.04	−0.50
Age	−.01	.01	−.09	−0.93	−.01	.01	−.06	−0.69
Tenure	−.01	.02	−.06	−0.66	−.02	.02	−.06	−0.64
Organization size	−.00	.00	−.02	−0.30	−.00	.00	−.03	−0.41

	*B*	SE	*β*	*t*(183)	*B*	SE	*β*	*t*(183)
Step 2								
Gender	.04	.08	.02	0.47	.02	.11	.01	0.17
Age	.00	.01	−.02	−0.22	.01	.01	.04	0.58
Tenure	.00	.01	.01	0.14	−.01	.02	−.03	−0.47
Organization size	.00	.00	−.01	−0.18	.00	.00	−.01	−0.21
Charismatic leadership	.06	.10	.04	0.62	−.23	.13	−.11	−1.71
Participative leadership	−.45	.10	−.34	−4.54^***^	−.03	.13	−.02	−0.25
Despotic leadership	.30	.06	.38	5.06^***^	.26	.08	.24	3.30^**^
Laissez-faire leadership	.22	.06	.24	3.64^***^	.70	.08	.55	8.58^***^

** *p* < .01; *** *p* < .001

### Leadership Styles and Job Insecurity

We conducted a similar hierarchical regression analysis on job insecurity. Results revealed that Step 1 was not significant, *F*(4, 187) = 0.72, *p* = .58, and that Step 2 was significant (Δ*R* ^*2*^ = .55), *F*(4, 183) = 56.65, *p* < .001. The full regression model was significant, (*R* ^*2*^ = .56), *F*(8, 183) = 29.11, *p* < .001. As can be seen in Table 2, only the destructive leadership styles (despotic and laissez-faire) significantly predicted job insecurity; the effects of the constructive leadership styles (charismatic and participative) were nonsignificant. Given that our theoretical model hinges on job insecurity as mediator between leadership styles and belief in conspiracy theories, these findings reveal that Hypothesis 4 is not supported by the data. In the following we therefore only include the destructive leadership styles in our linear structural model (consistent with the regression results presented here, a model that included the constructive leadership styles did not have an adequate fit).

### Linear Structural Model

Structural Equation Modeling (Arbuckle 2012) was used to integrate the relations between the leadership predictors, conspiracy beliefs, and the organizational outcome variables into a mediation model. First, based on our theoretical framework, we constructed a model in which we used the leadership variables as predictors, job insecurity as a first mediator, conspiracy beliefs as a second mediator, and organizational commitment and turnover intentions as criteria. In this model, we included all paths from the predictors to the two mediators and from the predictors and two mediators to the two criteria. Because the background variables were unrelated to job insecurity and conspiracy beliefs, we omitted them from our model.^4^ Second, for each of the variables in the model we constructed two parallel parcels. Although being debated (Marsh et al. 2013), the use of parcels (i.e., the combination of multiple items in one manifest variable) offers a number of practical and psychometric advantages, such as a reduction of error and unique variance in parcels when compared to items, and thus a more efficient representation of the construct space (Little et al. 2013). For two variables, job insecurity and turnover intentions, each parcel consisted of only one item. For all other variables, we employed the following technique to obtain the parcels: (a) on each of the constructs we conducted a Principal Component Analysis from which we extracted the first unrotated factor^5^ and (b) we included the items with the highest and the lowest loading on the first unrotated factor in the first parcel and we included the items with the one-but-highest and one-but-lowest loadings on the first unrotated factor in the second parcel, then again the items with the next-highest and next-lowest loadings in the first parcel, and so on. Third, we used conventional indices to ascertain the fit of the model, e.g., *χ* ^2^ with *p* > .05, comparative fit index (CFI), and root square error of approximation (RMSEA).

This first model, which had excellent fit (*χ* ^2^(39) = 34.00, *p* = .70; CFI = 1.00; RMSEA = .00), confirmed many of the expected relations of the predictors with the mediators, and of the mediators with the criteria (Table 3, left side columns). That is, laissez-faire leadership and despotic leadership were positively related to job insecurity, which in turn was positively related to conspiracy beliefs. Conspiracy beliefs was negatively related to organizational commitment, which—in line with previous research (Meyer et al. 2002)— was negatively related to turnover intentions. Two additional paths were significant, i.e., a path from despotic leadership to conspiracy beliefs and a path from job insecurity to turnover intentions.

**Table 3:** Structural models of the direct relations between the predictors (Despotic leadership and Laissez-Faire leadership), mediators (Job Insecurity and Conspiracy Beliefs), and criteria (Organizational Commitment and Turnover Intentions)

	Structural model with all relations				Final structural model			
	*B*	*SE*	β	*p*	*B*	*SE*	β	*p*
Despotic leadership → job insecurity	.23	.09	.12	.01	.25	.09	.24	<.01
Despotic leadership → conspiracy beliefs	.44	.07	.56	<.01	.43	.07	.54	<.01
Despotic leadership → Org. commitment	.02	.10	.03	.82			–	
Despotic leadership → turnover intentions	.12	.13	.10	.37			–	
Laissez-faire → job insecurity	.86	.12	.68	<.01	.84	.12	.68	<.01
Laissez-faire → conspiracy beliefs	−.05	.14	−.06	.70			–	
Laissez-faire → org. commitment	.25	.17	.28	.15	.20	.09	.23	.03
Laissez-faire → turnover intentions	.18	.23	.12	.44			–	
Job insecurity → conspiracy Beliefs	.23	.11	.30	.05	.20	.07	.26	<.01
Job insecurity → org. commitment	−.06	.15	−.09	.66			–	
Job insecurity → turnover intentions	.49	.19	.41	.01	.76	.08	.62	<.01
Conspiracy beliefs → org. commitment	−.40	.12	−.43	<.01	−.41	.10	−.44	<.01
Conspiracy beliefs → turnover intentions	.07	.16	.04	.68			–	
Org. commitment → turnover intentions	−.68	.12	−.40	<.01	−.71	.11	−.42	<.01

**Fig. 1:**
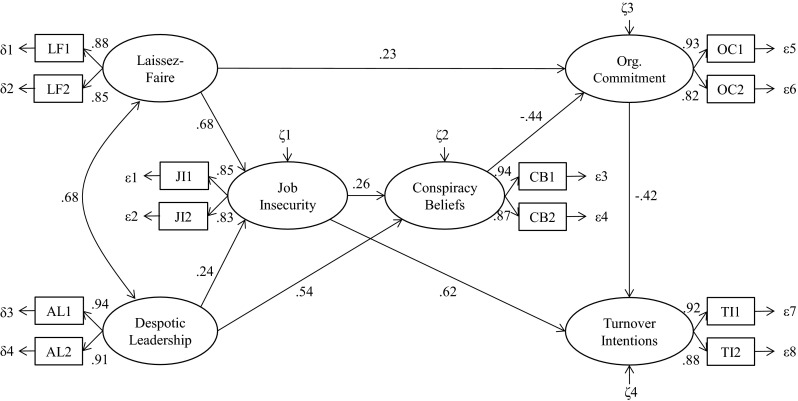
Final structural model of the relations between leadership styles, organizational conspiracy beliefs, and organizational variables. All standardized path coefficients are significant at *p* < .05. Model fit: *χ* ^2^(45) = 37.10, *p* = .79; CFI = 1.00; RMSEA = .00

We checked whether we could make this model more parsimonious by deleting—one by one—the weakest nonsignificant paths. We continued until all path coefficients were significant and no further improvement could be made without a significant deterioration of fit. This resulted in the final model, which is presented in Fig. 1. This final model was not significantly worse—and thus preferable, because more parsimonious—than the first model (Δ*χ* ^2^(6) = 3.10, *p* = .80; final model *χ* ^2^(45) = 37.10, *p* = .79; CFI = 1.00; RMSEA = .00). Using a bootstrap procedure in AMOS with 5,000 samples and a 95 % bias-correct confidence interval (CI) on this final model, the standardized parameters of the indirect effects showed that laissez-faire leadership had a significant indirect relation, via job insecurity, to conspiracy beliefs (*γ* = .18; CI = .03, .37). Furthermore, laissez-faire leadership had a significant indirect relation with turnover intentions through job insecurity, conspiracy beliefs, and organizational commitment (*γ* = .36; CI = .15, .55). Despotic leadership had a significant direct relation with conspiracy beliefs (*γ* = .54; CI = .30, .74) and via job insecurity, a significant indirect relation with conspiracy beliefs (*γ* = .06; CI = .00, .21). The total indirect relation of despotic leadership with turnover intentions through job insecurity, conspiracy beliefs, and organizational commitment was also significant (*γ* = .26; CI = .08, .45). The indirect relation of conspiracy beliefs with turnover intentions through organizational commitment was also significant (*γ* = .18; CI = .06, .32). In total, 74 % (*p* < .01) of the variance was explained by the two leadership variables in job insecurity, 56 % (*p* < .01) of the variance was explained by the two leadership variables and job insecurity in conspiracy beliefs, 13 % (*p* < .01) of the variance was explained in organizational commitment, and 60 % (*p* < .01) of the variance was explained in turnover intentions.

In sum, these results support Hypothesis 2: Feelings of job insecurity mediate the path from laissez-faire leadership to conspiracy beliefs and from despotic leadership to organizational conspiracy beliefs. The model furthermore offers support for the relation between conspiracy beliefs and organizational commitment (Hypothesis 5a) and reveals qualified support of Hypothesis 5b by revealing an indirect relationship between organizational conspiracy beliefs and turnover intentions, mediated by organizational commitment.

## Discussion

Whereas belief in conspiracy theories has been shown to be widespread in the context of citizen’s perceptions of macro-political and societal events (Oliver and Wood 2014; Sunstein and Vermeule 2009), the role of such beliefs in the micro-level setting of organizations has not yet been recognized. The present study was designed to establish if conspiracy beliefs are relevant to predict a number of variables that are essential to the proper functioning of organizations. The findings of our study clearly suggest that how employees perceive their leaders predict conspiracy beliefs. Whereas participative leadership is associated with decreased organizational conspiracy beliefs, despotic and laissez-faire leadership are associated with increased organizational conspiracy beliefs. Moreover, the relationship between laissez-faire leadership and organizational conspiracy beliefs is mediated by an increase in feelings of job insecurity. Furthermore, organizational conspiracy beliefs have implications for organizational outcomes, as they predict a decreased commitment to the organization among employees, and—through this decreased commitment—the extent to which they intend to resign their jobs. Taken together, these findings provide a first step toward establishing the importance of conspiracy beliefs to understand the functioning of employees within organizations.

The present study offers three novel conceptual insights. First, organizational conspiracy beliefs emerge frequently among employees when making sense of the behavior of their management. In fact, we approached a random sample of the US population, and presumably most (if not all) of our participants work in different organizations—hence, the organizational conspiracy beliefs that we observed are not specific to one particular (and potentially truly corrupt) organization. Yet, looking at Table 1, the mean of organizational conspiracy beliefs is slightly above the scale midpoint of 3.0. Across the sample, participants indicate being moderately open to the possibility that there are malevolent conspiracies within their organization, suggesting that such beliefs are meaningful to understand employees’ perceptions, emotions, and behaviors on the work floor. These relatively high levels match the observation that societal conspiracy theories are prevalent among citizens (Oliver and Wood 2014). It thus seems that people endorse conspiracy theories in various life domains suggesting a natural tendency for people to be suspicious of powerful groups. Such suspiciousness is particularly likely to emerge in situations that have personal relevance for perceivers, such as when a powerful group of managers initiates change that affects the lives of perceivers and co-workers that they connect their identity to (cf. Herold et al. 2008; Van Prooijen and Van Dijk 2014).

A second novel conceptual insight is that differences in perceived leadership styles are associated with organizational conspiracy beliefs. In our study, we focused on both constructive and destructive leadership styles, as these styles may influence feelings of job insecurity. Based on theoretical and empirical insights illuminating the role of uncertainty in conspiracy beliefs (Hofstadter 1966; Newheiser et al. 2011; Sullivan et al. 2010; Van Prooijen and Jostmann 2013; Whitson and Galinsky 2008), such job insecurity should be reflected in organizational conspiracy beliefs. The results partly supported this line of reasoning, underscoring that participative, despotic, and laissez-faire leadership have implications for organizational conspiracy beliefs, and that for despotic and laissez-faire leadership this role is explained by job insecurity. Third, organizational conspiracy beliefs are harmful to organizations. We specifically focused on two employee outcomes that are important to the functioning of organizations, notably organizational commitment, and turnover intentions. The data revealed that organizational conspiracy beliefs were detrimental to employees’ feelings of commitment, and indirectly also impacted participants’ turnover intentions.

The observation that conspiracy beliefs are harmful to organizations, in conjunction with the observation that many employees endorse organizational conspiracy beliefs to some extent, suggest an important practical implication of the present findings: Organizational conspiracy beliefs are not innocent rumors on the work floor that are safe for managers to ignore, but can have real and tangible consequences for employees, and hence, for organizations. We speculate about two possible interventions that may be promising when trying to reduce conspiracy beliefs in organizations. First, carefully implementing procedural justice principles in decision-making processes is likely to reduce the potential for suspicion about possible conspiracy formation. For instance, granting employees voice about relevant decisions improves their relationship with decision-makers (Tyler and Blader 2003) and provides them with a sense of autonomy (Van Prooijen 2009). Indeed, participative leadership is closely associated with the procedural justice principle of voice (Furst and Cable 2008), and our findings suggest that this leadership style reduces organizational conspiracy beliefs. A second possible intervention is to educate employees about the complexity of managerial decisions in a competitive market, and to thoroughly inform them why certain decisions were made—particularly in the case of decisions that are unpleasant for specific employees, but that may be necessary for the organization’s long-term collective interest (cf. Brockner et al. 1990). Recognizing that there often are no simple solutions to complex collective problems is associated with reduced conspiracy beliefs (Van Prooijen et al. 2015). More generally, an important avenue for future research is what interventions leaders can implement to reduce organizational conspiracy beliefs among their employees.

A potential methodological contribution of the present study is the novel measure of organizational conspiracy beliefs. To the best of our knowledge, no measurement instrument yet existed to assess this construct. Our scale has good reliability, and relates to job insecurity in ways that should be predicted based on previous insights (Hofstadter 1966; Van Prooijen and Jostmann 2013; Whitson and Galinsky 2008). These are preliminary indications that our scale might be a measurement instrument with high construct validity. Nevertheless, we urge to note that this study was not designed as a validation study, and the specific validity of our organizational conspiracy belief as a generic measurement tool, that is applicable to a multitude of research questions within an organizational context, remains an open question. Hence, the main contributions of the study are the conceptual points mentioned above. Future research would do well to more thoroughly validate the organizational conspiracy belief scale that we developed for the present purposes.

Our main propositions are independent from the question whether or not there may be a grain of truth in certain conspiracy theories. Corruption and power-abuse does occur in organizations, and sometimes employees are right to be suspicious. In fact, it has been noted that people may be suspicious of their leaders either because these leaders actually are corrupt; or, because of exaggerated suspicion and paranoia among employees; or, because of a combination of both—the latter referring to situations where leaders are not fully honest, but employees at the same time overestimate leaders’ evil intentions (Van Prooijen and Van Lange 2014). The model presented here merely reflects employees’ subjective beliefs, and does not exclude the possibility that, sometimes, their beliefs are correct and reflect actual conspiracies within the organization. Indeed, an interesting open question is to what extent certain leadership styles are associated with actual corruption and conspiracy formation. To what extent are the leadership styles that were under investigation here diagnostic for the likelihood that supervisors or managers truly are involved in conspiracies? Future research may address this issue.

## Limitations

Our findings were based on a random sample of the US working population, including many different organizations. In some respects this organizational heterogeneity is a strength, as it suggests that our conclusions are not restricted to one type of industry. At the same time, this issue also suggests an important avenue for further research. Different types of industry are likely to have their own norms about appropriate leader behavior: For instance, a despotic leader may be considered more normative in the army than in other types of organization. Likewise, a laissez-faire leader is more likely to be accepted in an academic setting than in organizations where success depends on carefully monitoring employees’ goals and activities. Although speculative, we do not expect type of industry to change the *direction* of the effects, as various other leadership effects have been found to replicate across organizations (e.g., see Britt et al. 2004, versus Dale and Fox 2008, for an illustration of leadership styles that have comparable effects on role stress in the army versus in a manufacturing company). Nevertheless, it is plausible that type of industry impacts the *relative strength* of the effects: For instance, the relationship between despotic leadership and organizational conspiracy beliefs may be relatively weak in types of industry where despotic leadership is the norm.

A methodological limitation of the present study is the fact that we used a cross-sectional design, leaving questions about causality, response bias, and common method variance. Note, however, that the model displayed in Fig. 1 is consistent with previous theorizing. Particularly the influence of job insecurity on organizational conspiracy beliefs has a strong theoretical basis, as the causal effects of uncertainty on belief in conspiracy theories have been shown in various studies (Sullivan et al. 2010; Van Prooijen, in press; Van Prooijen and Jostmann 2013; Whitson and Galinsky 2008). Moreover, concerns about possible response bias and common method variance are alleviated by two complementary observations: (1) the four leadership variables, and belief in conspiracy theories, all loaded on different factors in a CFA, suggesting that participants conceptually distinguished between these constructs, and (2) whereas some leadership styles exerted the predicted effects, other leadership styles did not influence organizational conspiracy beliefs (e.g., charismatic leadership). Furthermore, many constructs that were central in our contribution were privately held beliefs, which necessarily rely on self-reports. Indeed, Conway and Lance (2010) note that for such privately held beliefs, cross-sectional self-reports can be acceptable and even necessary, provided that a few conditions are met (i.e., good construct validity of the scales; lack of scale overlap; and a solid questionnaire that minimizes the concerns associated with self-reports). These considerations notwithstanding, we do not claim to have resolved the methodological concerns that are associated with cross-sectional designs in the present study. Instead, we hope that the present findings may provide a starting point for a novel line of research on the causes and consequence of conspiracy beliefs in organizations. Future research needs to complement the current findings with more sophisticated research designs, that are based on multiple source data, or on longitudinal measurements.

## Concluding Remarks

Inspired by the observation that conspiracy beliefs are widespread in citizens’ perceptions of macro-level societal events, in the present contribution we posed the question whether conspiracy belief is also an important variable to consider when studying perceptions and behaviors of employees in an organizational setting. The results of the current study provide an affirmative answer to this question. Furthermore, by empirically connecting organizational conspiracy beliefs to the psychology of leadership, the study presented here suggests a new perspective on leader effectiveness as represented in the influence of various leadership styles on employee outcomes. Disapproving of some of the decisions that a supervisor makes is one thing, suspecting a supervisor to be involved in evil conspiracies is quite another issue, with far-reaching implications. We conclude that organizational conspiracy beliefs are prevalent among employees, and have substantial implications for leadership styles and organizational outcomes.

## Footnotes

1 In the study of Redeker et al. (2014), participative leadership was only moderately ‘passive’; however, it is substantially more passive than charismatic leadership, a constructive leadership style that is regarded as very active.

2 Initially our scale comprised of 12 items, but following reviewer recommendations, a content validity review led us to drop three items (i.e., “Our managers try to minimize the investment in us,” “our managers try to maximize profits at our expense,” and “Nepotism (friend politics) is a practice which is present at work”). These items may be regarded as unsupportive leadership behaviors, and do not necessarily entail the element of secrecy that is inherent to conspiracy beliefs. If these items were included in the scale, results were similar.

3 Because the four leadership styles and organizational conspiracy beliefs all assessed employees’ perceptions of their leaders, we first conducted a confirmatory factor analysis using all items of these five scales in which we allowed the factors to covary. Taking into account the large number of degrees of freedom, the model showed a reasonable fit (*χ* ^2^(584) = 1248.05, *p* *<* .01; CFI = .81; RMSEA = .08). All of the items had a significant loading on the intended factors apart from the two reverse-coded conspiracy belief items, whose loadings were in the right direction, but not significant. Exclusion of these two items did not improve the reliability of the organizational conspiracy belief scale appreciably (i.e., Cronbach’s alpha improves from .87 to .92). Given that the wording of these items suggests high construct validity, and given the methodological value of having reverse-scored items, we decided to retain both these items in our scale of organizational conspiracy beliefs.

4 Note that age and tenure were related to turnover intentions. Adding either one of them in the final model resulted in only weak relations of age or tenure to turnover intentions, so we decided to omit them from the model.

5 Most of the Principal Component Analyses showed that the constructs were unidimensional. In a few cases, deviations from multidimensionality were observed. However, we surmised—based on the original research on the constructs in question that indicated unidimensionality—that this may have been due to sampling fluctuations and thus we decided to stick to the unidimensional solution for the construction of the parcels.
